# ProtSeqGen: a novel deep learning model for protein sequence design

**DOI:** 10.1186/s12859-026-06482-4

**Published:** 2026-05-26

**Authors:** Qiang Gao, Zhijin Li, Yang Deng, Zhiwei Ji

**Affiliations:** 1 College of Artificial Intelligence, Nanjing Agricultural University, No. 666 Binjiang Avenue, Nanjing, 211800 Jiangsu China; 2 Center for Data Science and Intelligent Computing, Nanjing Agricultural University, No. 666 Binjiang Avenue, Nanjing, 211800 Jiangsu China; 3 Department of Neurosurgery, Division of Life Science and Medicine, The First Affiliated Hospital of USTC (Anhui Provincial Hospital), University of Science and Technology of China, Hefei, 230036 Anhui China; 4 School of Life Science and Technology, Harbin Institute of Technology, Harbin, 150001 Heilongjiang China

**Keywords:** Protein design, Backbone, Sequence, Residue, Recovery

## Abstract

The protein inverse folding problem, which is the task of designing an amino acid sequence that will fold into a specified backbone structure, represents a fundamental challenge in *de novo* protein design. Existing computational methods, including deep learning-based approaches, often fail to simultaneously optimize accuracy, stability, efficiency, and generalizability across diverse folds. Here, we present ProtSeqGen, a deep learning model that overcomes these limitations through a multi-stage graph-based framework. ProtSeqGen encodes protein structures as local geometric graphs, explicitly models residue-level interactions using a message-passing neural network, and predicts optimal amino acids with a multi-layer perceptron. When trained on CATH 4.2 dataset and evaluated on standard and challenging benchmarks, ProtSeqGen achieved superior sequence recovery compared to numerous state-of-the-art (SOTA) methods. It also generated accurate, designable sequences for nine topologically diverse proteins, demonstrating remarkable generalization capability. These results establish ProtSeqGen as a robust and scalable solution to the protein inverse folding problem, propelling *de novo* protein design with high structural precision.

**Supplementary Information:**

The online version contains supplementary material available at 10.1186/s12859-026-06482-4.

## Introduction

Proteins are essential biological macromolecules that carry out diverse catalytic, regulatory, and structural roles through their precisely folded three-dimensional conformations, which are determined by their specific amino acid sequences [1–3]. A central objective in molecular biology is the *de novo* design of functional protein sequences, a task also known as the ​protein inverse folding problem, with broad applications in therapeutic development, enzyme engineering, and synthetic biology [4, 5]. Conventional computational approaches typically formulate this task as an energy minimization problem, using physical force fields and conformational sampling to identify low-energy sequences for a given backbone [6, 7]. However, these methods are limited by inaccuracies in energy functions and the exponential complexity of sequence space, constraining their robustness and scalability [8]. Although recent AI-based strategies have advanced protein design, developing computational frameworks that are simultaneously accurate, efficient, and generalizable across diverse structural motifs remains a significant challenge [9].

Conventional computational approaches for protein design can be broadly categorized as physics-based or statistics-based. Physics-based methods formulate inverse folding as an energy minimization problem, using molecular force fields to identify amino acid sequences that stabilize a given backbone structure [10, 11]. Their performance, however, is often limited by inaccuracies in energy functions and the high computational cost of sampling large sequence spaces. In contrast, statistic-based approaches infer sequence-structure relationships from multiple sequence alignments or structural databases, but they tend to generalize poorly to novel folds and perform particularly poorly in data-scarce settings [7, 12]. Consequently, both paradigms face challenges in achieving accurate and efficient *de novo* protein sequence design.

Beyond conventional physics- and statistics-based strategies, machine learning (ML) techniques have also been applied to protein sequence prediction and classification. Methods such as Support Vector Machines (SVM) and Naive Bayes classifiers were used to distinguish high- and low-designability sequences based on binary hydrophobic/polar (H/P) patterns and lattice-model energetics [13]. Additionally, neural network-based frameworks such as SPIN integrated local structural fragments with energy information to directly predict backbone-compatible sequences, outperforming traditional fragment-assembly methods in both accuracy and efficiency [14]. Collectively, these early ML approaches demonstrated the feasibility of data-driven modeling in capturing sequence-structure relationships and laid the conceptual foundation for modern deep learning-based methods.

Recent advances in deep learning have enabled more effective modeling of sequence-structure relationships directly from protein backbone geometries [15, 16]. Early efforts employed 3D convolutional networks (CNNs), such as ProDCoNN, to capture multi-scale spatial features, albeit with substantial computational overhead [17]. Subsequent approaches, including SPROF, improved computational efficiency by representing 3D structures as 2D residue-residue distance maps, though this transformation resulted in partial loss of geometric information [18]. More recently, graph-based methods have become the dominant paradigm, representing protein structures as graphs where residues serve as nodes and spatial interactions as edges, thereby naturally capturing non-local structural contexts. A seminal study by Ingraham et al.. reformulated the inverse folding problem as a structure-to-sequence translation task, introducing an encoder-decoder architecture that uses local reference frames and backbone dihedrals to represent geometric features, and proposed two encoder variants: StructTrans and StructGNN [19]. Subsequent models have extended this framework with diverse architectural and training innovations. GVP-GNN jointly processes scalar and geometric features within graph representations [20], while ProteinMPNN enhances sequence recovery rates by incorporating full atomic coordinates and edge updates [21]. PiFold captures multi-scale residue interactions by combining a featurizer with a stacked PiGNN [22], and GeoSeqBuilder integrates all-atom representations with three-body residue modeling to enhance structural fidelity [23]. Further innovations leverage large-scale pretraining, such as ESM-IF, which uses millions of AlphaFold3-predicted structures [24], and generative frameworks like AlphaDesign, which combines AlphaFold with diffusion models for controllable protein design [25]. Overall, these methods form constitute an increasingly effective toolkit for the protein inverse folding problem.

**Fig. 1:**
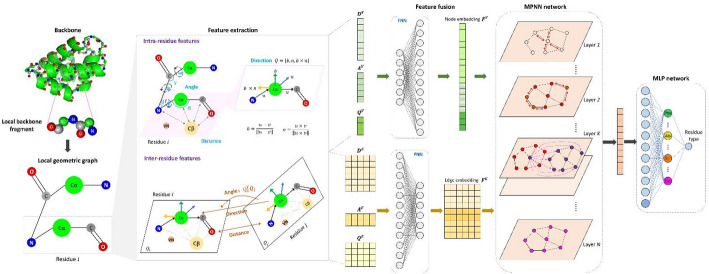
The whole architecture of ProtSeqGen

In this study, we introduce ProtSeqGen, an end-to-end deep learning model for protein sequence design from 3D backbone structures (Fig. 1). The model employs a multi-stage representation learning strategy, in which each input structure is encoded as local geometric graphs to extract intra-residue vertex and inter-residue edge features. These features are then fused and optimized through a Message-Passing Neural Network (MPNN) to capture critical structural constraints, followed by a Multi-Layer Perceptron (MLP) that predicts the most probable amino acid residue at each position. Our model was trained on CATH 4.2 dataset and evaluated on standard benchmarks (TS50 and TS500) as well as challenging benchmarks such as IDRome-120 (Intrinsically Disordered Regions [26, 27], IDRs) and TS45 (Free modeling (FM) targets [28]). ProtSeqGen demonstrated superior performance and generalization capability compared to a comprehensive suite of state-of-the-art (SOTA) models. Evaluated on nine structurally diverse proteins, ProtSeqGen demonstrated robust *de novo* design capability, generating sequences that folded into native-like backbone conformations​ and exhibited superior structural stability. Collectively, these results establish ProtSeqGen as a versatile tool for designing novel sequences for predefined protein structures with atomic details.

## Results

### Overview of ProtSeqGen

The overall architecture of ProtSeqGen is illustrated in Fig. 1. The model takes a protein backbone as input and generates an amino acid sequence predicted to fold into the given structure. The computational pipeline consists of five sequential stages designed to enable high-accuracy sequence generation, including (1) generating local geometric graphs; (2) extracting residue-scale features of ‘node’ and ‘edge’; (3) integrating residue representation via feature fusion; (4) refining features via an MPNN network; and (5) predicting residue types using an MLP classifier. ProtSeqGen was trained on the CATH 4.2 dataset and evaluated on both standard benchmarks (TS50 and TS500) and more challenging benchmarks (IDRome-120 and TS45) (Table 1, Supplementary Table 1).

**Table 1:** Description of datasets used in this study

Datasets	Training	Validation	Testing
CATH 4.2	18,024	608	1,120
TS500	–	–	500
TS50	–	–	50
IDRome-120	–	–	120
TS45	–	–	45

###  Ablation studies on model architecture

To validate the design of our model architecture, we systematically dissected the contributions of structural configurations, core components, and feature representations through comprehensive ablation studies.

Firstly, we assessed structural choices by evaluating various combinations of $\:C\beta\:$ geometry and virtual nodes. As shown in Table 2, incorporating $\:C\beta\:$ geometry was pivotal, with the model achieving peak performance​ specifically when utilizing 2 virtual nodes.

**Table 2:** Ablation study on the impact of $\:C\beta\:$ geometry and virtual node configuration on model performance. The **best** results are highlighted in bold.

Condition	Virtual node	Recovery (%) $\:\uparrow\:$	Perplexity $\:\downarrow\:$	Loss $\:\downarrow\:$
With $\:C\beta\:$ geometry	0	51.39	4.58	1.47
	1	52.19	4.49	1.46
	2	**53.03**	**4.43**	**1.45**
	3	52.66	4.46	1.45
Without $\:C\beta\:$ geometry	0	49.71	4.79	1.51
	1	50.41	4.64	1.48
	2	51.38	4.57	1.47
	3	50.95	4.62	1.48

**Table 3:** Ablation study on two feature groups. The **best** results are highlighted in bold.

Model variant	Intra-residue features	Inter-residue features	Attention in MPNN	Recovery (%) $\:\uparrow\:$	Perplexity $\:\downarrow\:$	Loss $\:\downarrow\:$
$\:{M}_{1}$	$\:\surd\:$	$\:\times\:$	$\:\times\:$	25.18	10.92	2.39
$\:{M}_{2}$	$\:\surd\:$	$\:\times\:$	$\:\surd\:$	27.59	10.38	2.33
$\:{M}_{3}$	$\:\times\:$	$\:\surd\:$	$\:\times\:$	44.35	5.98	1.79
$\:{M}_{4}$	$\:\times\:$	$\:\surd\:$	$\:\surd\:$	46.12	5.59	1.73
$\:{M}_{5}$	$\:\surd\:$	$\:\surd\:$	$\:\times\:$	49.86	5.03	1.61
**ProtSeqGen**	$\:\surd\:$	$\:\surd\:$	$\:\surd\:$	**53.03**	**4.43**	**1.45**

Secondly, we investigated the functional modules by quantifying the impact of intra-residue versus inter-residue features (Table 3). While inter-residue features provide the primary driving force for sequence generation, the synergistic fusion of both feature groups is indispensable for optimal accuracy.

Finally, we performed a leave-one-out analysis on all six distinct feature types ($\:{D}^{V}$, $\:{A}^{V}$, $\:{Q}^{V}$, $\:{D}^{E}$, $\:{A}^{E}$, and $\:{Q}^{E}$) (see Fig. 1), to pinpoint their individual importance. As evidenced by Table 4, the removal of any inter-residue feature resulted in a marked degradation of performance (e.g., a substantial decrease in recovery), underscoring their collective necessity for accurate prediction.

**Table 4:** Ablation study on individual features. The **best** results are highlighted in bold.

Model variant	Recovery (%) $\:\uparrow\:$	Perplexity $\:\downarrow\:$	Loss $\:\downarrow\:$
Without $\:{D}^{V}$	49.91	4.76	1.54
Without $\:{A}^{V}$	51.09	4.72	1.54
Without $\:{Q}^{V}$	51.17	4.71	1.53
Without $\:{D}^{E}$	43.65	6.04	1.69
Without $\:{A}^{E}$	48.27	4.95	1.61
Without $\:{Q}^{E}$	49.84	4.88	1.58
**ProtSeqGen**	**53.03**	**4.43**	**1.45**

Collectively, these results confirm the validity of the proposed architectural design.

### Evaluating ProtSeqGen against SOTA methods on CATH 4.2

On the CATH 4.2 testing set, ProtSeqGen demonstrates superior performance when benchmarked against a comprehensive set of SOTA methods.​​ As summarized in Table 5, our model achieves a ​sequence recovery rate of 53.03%​, outperforming all other models. In particular, ProtSeqGen improves recovery by ​7.3% over the widely adopted ProteinMPNN (45.74%), and also surpasses strong graph-based competitors such as PiFold (51.69%) and GeoSeqBuilder (51.89%) by margins ​1.3%​​ and ​1.1%​, respectively. Furthermore, ProtSeqGen achieves the lowest perplexity (4.43) and loss (1.45), reflecting higher prediction confidence and accuracy. Although other SOTA methods, including Frame2seq and AlphaDesign, also exhibit competitive results, ProtSeqGen consistently achieves top performance across all key metrics, highlighting the effectiveness of its novel local geometric graph representation and feature fusion strategy.

**Table 5:** Performance evaluation on CATH 4.2 dataset (mean $\:\pm\:$ SD). The** best** results are highlighted in bold.

Feature Embedding	Method	Recovery (%) $\:\uparrow\:$	Perplexity $\:\downarrow\:$	Loss $\:\downarrow\:$
MLP	SPIN	28.68 $\:\pm\:$ 0.25	9.17 $\:\pm\:$ 0.12	2.33 $\:\pm\:$ 0.03
	SPIN2	30.84 $\:\pm\:$ 0.26	8.74 $\:\pm\:$ 0.09	2.21 $\:\pm\:$ 0.03
	Wang’s model	30.93 $\:\pm\:$ 0.23	8.62 $\:\pm\:$ 0.11	2.16 $\:\pm\:$ 0.02
CNN	SPROF	34.09 $\:\pm\:$ 0.19	7.37 $\:\pm\:$ 0.10	1.96 $\:\pm\:$ 0.04
	ProDCoNN	34.13 $\:\pm\:$ 0.23	7.24 $\:\pm\:$ 0.08	1.91 $\:\pm\:$ 0.03
	DenseCPD	43.69 $\:\pm\:$ 0.27	5.83 $\:\pm\:$ 0.05	1.52 $\:\pm\:$ 0.04
GNN	StructGNN	35.64 $\:\pm\:$ 0.24	6.71 $\:\pm\:$ 0.07	1.86 $\:\pm\:$ 0.03
	GraphTrans	35.95 $\:\pm\:$ 0.18	6.39 $\:\pm\:$ 0.08	1.78 $\:\pm\:$ 0.02
	GVP	37.63 $\:\pm\:$ 0.17	6.05 $\:\pm\:$ 0.06	1.67 $\:\pm\:$ 0.02
	GCA	39.89 $\:\pm\:$ 0.21	5.32 $\:\pm\:$ 0.07	1.61 $\:\pm\:$ 0.03
	AlphaDesign	41.34 $\:\pm\:$ 0.22	5.11 $\:\pm\:$ 0.07	1.58 $\:\pm\:$ 0.02
	ProteinMPNN	45.74 $\:\pm\:$ 0.10	4.61 $\:\pm\:$ 0.05	1.54 $\:\pm\:$ 0.01
	Frame2seq	49.08 $\:\pm\:$ 0.15	4.58 $\:\pm\:$ 0.06	1.50 $\:\pm\:$ 0.02
	PiFold	51.69 $\:\pm\:$ 0.09	4.55 $\:\pm\:$ 0.04	1.48 $\:\pm\:$ 0.01
	GeoSeqBuilder	51.89 $\:\pm\:$ 0.12	4.53 $\:\pm\:$ 0.05	1.48 $\:\pm\:$ 0.01
	**ProtSeqGen**	**53.03** $\:\pm\:$ **0.07**	**4.43** $\:\pm\:$ **0.03**	**1.45** $\:\pm\:$ **0.01**

### Generalization evaluation on canonical benchmarks: TS50 and TS500

To evaluate the generalization ability of ProtSeqGen, we assessed the pre-trained model on two independent testing sets: TS50 and TS500, which contain 50 and 500 unseen protein samples, respectively. As summarized in Table 6 and 7, ProtSeqGen achieved recovery rates of 58.51% (TS50) and 60.59% (TS500), outperforming most SOTA models. Notably, it significantly exceeds strong competitors like ProteinMPNN (54.43% on TS50, 58.08% on TS500). On average, ProtSeqGen demonstrates marginally higher accuracy compared to​ PiFold and GeoSeqBuilder.

**Table 6:** Performance evaluation on unseen TS50 samples using models trained on CATH 4.2 (mean $\:\pm\:$ SD). The **best** results are highlighted in bold.

Feature Embedding	Method	Recovery (%) $\:\uparrow\:$	Perplexity $\:\downarrow\:$	Loss $\:\downarrow\:$
MLP	SPIN	30.12 $\:\pm\:$ 0.43	9.18 $\:\pm\:$ 0.16	2.33 $\:\pm\:$ 0.05
	SPIN2	33.45 $\:\pm\:$ 0.39	8.52 $\:\pm\:$ 0.14	2.18 $\:\pm\:$ 0.04
	Wang’s model	32.93 $\:\pm\:$ 0.41	8.70 $\:\pm\:$ 0.15	2.21 $\:\pm\:$ 0.05
CNN	SPROF	39.13 $\:\pm\:$ 0.33	7.42 $\:\pm\:$ 0.12	1.96 $\:\pm\:$ 0.03
	ProDCoNN	40.79 $\:\pm\:$ 0.36	7.20 $\:\pm\:$ 0.11	1.89 $\:\pm\:$ 0.04
	DenseCPD	50.27 $\:\pm\:$ 0.29	5.87 $\:\pm\:$ 0.09	1.54 $\:\pm\:$ 0.03
GNN	StructGNN	43.75 $\:\pm\:$ 0.44	5.38 $\:\pm\:$ 0.08	1.45 $\:\pm\:$ 0.02
	GraphTrans	42.08 $\:\pm\:$ 0.32	5.67 $\:\pm\:$ 0.09	1.51 $\:\pm\:$ 0.03
	GVP	43.98 $\:\pm\:$ 0.39	5.34 $\:\pm\:$ 0.08	1.43 $\:\pm\:$ 0.02
	GCA	47.10 $\:\pm\:$ 0.26	4.78 $\:\pm\:$ 0.07	1.32 $\:\pm\:$ 0.02
	AlphaDesign	48.22 $\:\pm\:$ 0.34	5.21 $\:\pm\:$ 0.11	1.47 $\:\pm\:$ 0.04
	ProteinMPNN	54.43 $\:\pm\:$ 0.18	3.93 $\:\pm\:$ 0.06	1.39 $\:\pm\:$ 0.02
	Frame2seq	56.32 $\:\pm\:$ 0.23	3.87 $\:\pm\:$ 0.06	1.35 $\:\pm\:$ 0.02
	PiFold	58.38 $\:\pm\:$ 0.21	3.82 $\:\pm\:$ 0.08	1.30 $\:\pm\:$ 0.03
	GeoSeqBuilder	57.97 $\:\pm\:$ 0.17	3.86 $\:\pm\:$ 0.06	1.32 $\:\pm\:$ 0.02
	**ProtSeqGen**	**58.51** $\:\pm\:$ **0.15**	**3.79** $\:\pm\:$ **0.05**	**1.29** $\:\pm\:$ **0.02**

**Table 7:** Performance evaluation on unseen TS500 samples using models trained on CATH 4.2 (mean $\:\pm\:$ SD). The **best** results are highlighted in bold.

Feature Embedding	Method	Recovery (%) $\:\uparrow\:$	Perplexity $\:\downarrow\:$	Loss $\:\downarrow\:$
MLP	SPIN	30.28 $\:\pm\:$ 0.39	9.05 $\:\pm\:$ 0.11	2.28 $\:\pm\:$ 0.04
	SPIN2	36.73 $\:\pm\:$ 0.34	8.32 $\:\pm\:$ 0.15	2.06 $\:\pm\:$ 0.03
	Wang’s model	36.21 $\:\pm\:$ 0.31	8.39 $\:\pm\:$ 0.12	2.11 $\:\pm\:$ 0.04
CNN	SPROF	40.14 $\:\pm\:$ 0.34	7.18 $\:\pm\:$ 0.10	1.89 $\:\pm\:$ 0.03
	ProDCoNN	42.05 $\:\pm\:$ 0.27	6.85 $\:\pm\:$ 0.13	1.82 $\:\pm\:$ 0.03
	DenseCPD	55.63 $\:\pm\:$ 0.22	5.67 $\:\pm\:$ 0.07	1.48 $\:\pm\:$ 0.02
GNN	StructGNN	45.59 $\:\pm\:$ 0.28	4.96 $\:\pm\:$ 0.09	1.34 $\:\pm\:$ 0.01
	GraphTrans	44.62 $\:\pm\:$ 0.26	5.13 $\:\pm\:$ 0.08	1.41 $\:\pm\:$ 0.03
	GVP	49.25 $\:\pm\:$ 0.19	4.13 $\:\pm\:$ 0.06	1.42 $\:\pm\:$ 0.02
	GCA	47.69 $\:\pm\:$ 0.22	4.79 $\:\pm\:$ 0.06	1.33 $\:\pm\:$ 0.02
	AlphaDesign	49.18 $\:\pm\:$ 0.23	4.88 $\:\pm\:$ 0.07	1.36 $\:\pm\:$ 0.01
	ProteinMPNN	58.08 $\:\pm\:$ 0.14	3.53 $\:\pm\:$ 0.04	1.32 $\:\pm\:$ 0.01
	Frame2seq	59.17 $\:\pm\:$ 0.16	3.49 $\:\pm\:$ 0.05	1.31 $\:\pm\:$ 0.02
	PiFold	60.42 $\:\pm\:$ 0.13	3.44 $\:\pm\:$ 0.04	1.28 $\:\pm\:$ 0.01
	GeoSeqBuilder	60.45 $\:\pm\:$ 0.15	3.43 $\:\pm\:$ 0.04	1.28 $\:\pm\:$ 0.01
	**ProtSeqGen**	**60.59** $\:\pm\:$ **0.11**	**3.37** $\:\pm\:$ **0.04**	**1.27** $\:\pm\:$ **0.01**

Moreover, we conducted a rigorous statistical analysis across TS50 and TS500 to compare ProtSeqGen against three representative methods (ProteinMPNN, PiFold, and GeoSeqBuilder) (Supplementary Tables 2 and 3). Our analysis confirms that ProtSeqGen achieves statistically significant superiority over the majority of these competing methods across most metrics. However, its advantage marginally falls short of​ statistical significance when compared to PiFold on TS50 ($\:p=0.0797$) and GeoSeqBuilder on TS500 ($\:p=0.188$), hinting at a narrow performance boundary in specific folding contexts.

### Per-residue accuracy and prediction confidence

**Fig. 2:**
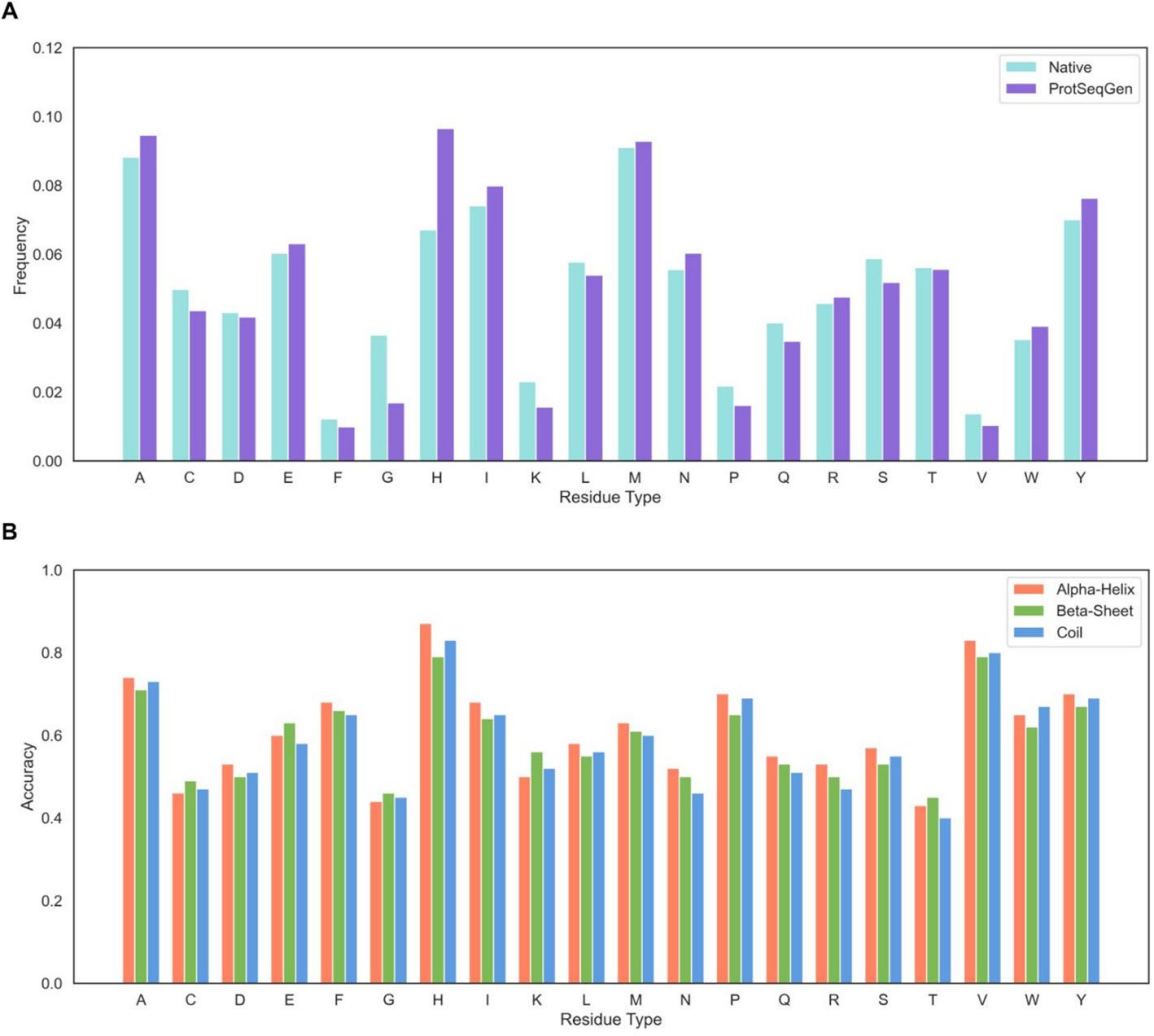
Impact of sequence redesign on *de novo* designed proteins (TS500). **A** The individual residue frequency between the native and predictions; **B** The predicted accuracy of residue types for three secondary structures

In this subsection, we evaluate the amino acid-specific prediction accuracy of ProtSeqGen on the TS500 dataset and compared its predictions with native proteins. ProtSeqGen exhibits a balanced preference for diverse residue types, including hydrophobic, aromatic, polar, and charged, and achieves secondary structure-dependent prediction accuracy aligned with known physicochemical propensities (Fig. 2A). Furthermore, we analyzed the differences in the central residue recovery rate across different secondary structures by calculating prediction accuracy per residue type within Alpha-Helix, Beta-Sheet, and Coil. Consistent with their secondary structural preferences, Cys (C), Lys (K), and Thr (T) were predicted more accurately in Beta-sheets, whereas His (H), Pro (P), and Tyr (Y) showed higher accuracy in Alpha -helices and coils, respectively (Fig. 2B). Detailed per-residue accuracy data are provided in Supplementary Tables 4 and 5.

### Generalization evaluation on challenging benchmarks: IDRome-120 and TS45

To assess the capability of our model on challenging tasks, we applied the pre-trained ProtSeqGen on specialized datasets featuring intrinsically disordered regions (IDRs) [26] and free-modeling (FM) targets [29] (Table 1).

First, we constructed the IDRoom-120 dataset, comprising 120 proteins of diverse sequence lengths from Tesei’s work (Supplementary Table 6, Text 1) [26]. As shown in Table 8, ProtSeqGen delivered significantly superior performance in generating intrinsically disordered sequences, while the predictive performance of all four methods decreased relative to TS50 and TS500.

**Table 8:** Performance evaluation on IDRome-120 and TS45 using models trained on CATH 4.2 (mean $\:\pm\:$ SD). The **best** results are highlighted in bold.

Dataset	Method	Recovery (%) $\:\uparrow\:$	Perplexity $\:\downarrow\:$	Loss $\:\downarrow\:$
IDRome-120	ProteinMPNN	31.83 $\:\pm\:$ 0.38	12.87 $\:\pm\:$ 0.21	3.39 $\:\pm\:$ 0.04
	PiFold	35.17 $\:\pm\:$ 0.19	9.35 $\:\pm\:$ 0.14	2.92 $\:\pm\:$ 0.03
	GeoSeqBuilder	36.52 $\:\pm\:$ 0.24	8.90 $\:\pm\:$ 0.12	2.81 $\:\pm\:$ 0.02
	**ProtSeqGen**	**41.41** $\:\pm\:$ **0.15**	**6.84** $\:\pm\:$ **0.03**	**1.92** $\:\pm\:$ **0.01**
TS45	ProteinMPNN	42.15 $\:\pm\:$ 0.27	8.41 $\:\pm\:$ 0.11	2.14 $\:\pm\:$ 0.02
	PiFold	48.97 $\:\pm\:$ 0.15	6.11 $\:\pm\:$ 0.02	1.66 $\:\pm\:$ 0.01
	GeoSeqBuilder	48.81 $\:\pm\:$ 0.12	6.06 $\:\pm\:$ 0.02	1.67 $\:\pm\:$ 0.01
	**ProtSeqGen**	**50.13** $\:\pm\:$ **0.08**	**5.09** $\:\pm\:$ **0.01**	**1.62** $\:\pm\:$ **0.01**

Second, we employed TS45, a highly challenging benchmark for *de novo* protein design consisting of FM targets with no homology to known structures [28, 30]. The results on TS45 mirror those on IDRome-120, underscoring the comparable difficulty of these two challenging benchmarks (Table 8).

### *De Novo* protein design with ProtSeqGen

In this study, we selected nine representative proteins to evaluate the capability of ProtSeqGen in generating amino acid sequences that accurately fold into their corresponding native three-dimensional structures (Table 9).​​ The selected proteins span a broad range of structural and functional diversity, including all-$\:\boldsymbol{\alpha\:}$, all-$\:\boldsymbol{\beta\:}$, $\:\boldsymbol{\alpha\:}/\boldsymbol{\beta\:}$, and $\:\boldsymbol{\alpha\:}+\boldsymbol{\beta\:}$ folds, as well as complex membrane proteins. Furthermore, the targets cover a gradient of structural complexity, ranging from simple motifs to intricate TIM-barrels [31], thereby providing a comprehensive and rigorous benchmark for assessing the model’s sequence generation quality.

**Table 9:** Detailed information of nine representative proteins for benchmarking

No.	PDB ID	Category	Subclass
2	1n27.A	$\:\alpha\:+\beta\:$	Zinc finger-like fold
3	1xe7.A	$\:\alpha\:/\beta\:$	TIM beta/alpha-barrel
4	1hzf.A	$\:\alpha\:+\beta\:$	Helix-turn-helix fold
6	1fvp.A	All-$\:\beta\:$	Jelly Roll
7	2qk2.A	Membrane Proteins	Voltage-gated ion channels-like superfamily
8	3u5t.A	$\:\alpha\:/\beta\:$	TIM beta/alpha-barrel
9	3h36.A	Membrane Proteins	​G protein-coupled receptor-like superfamily

**Fig. 3:**
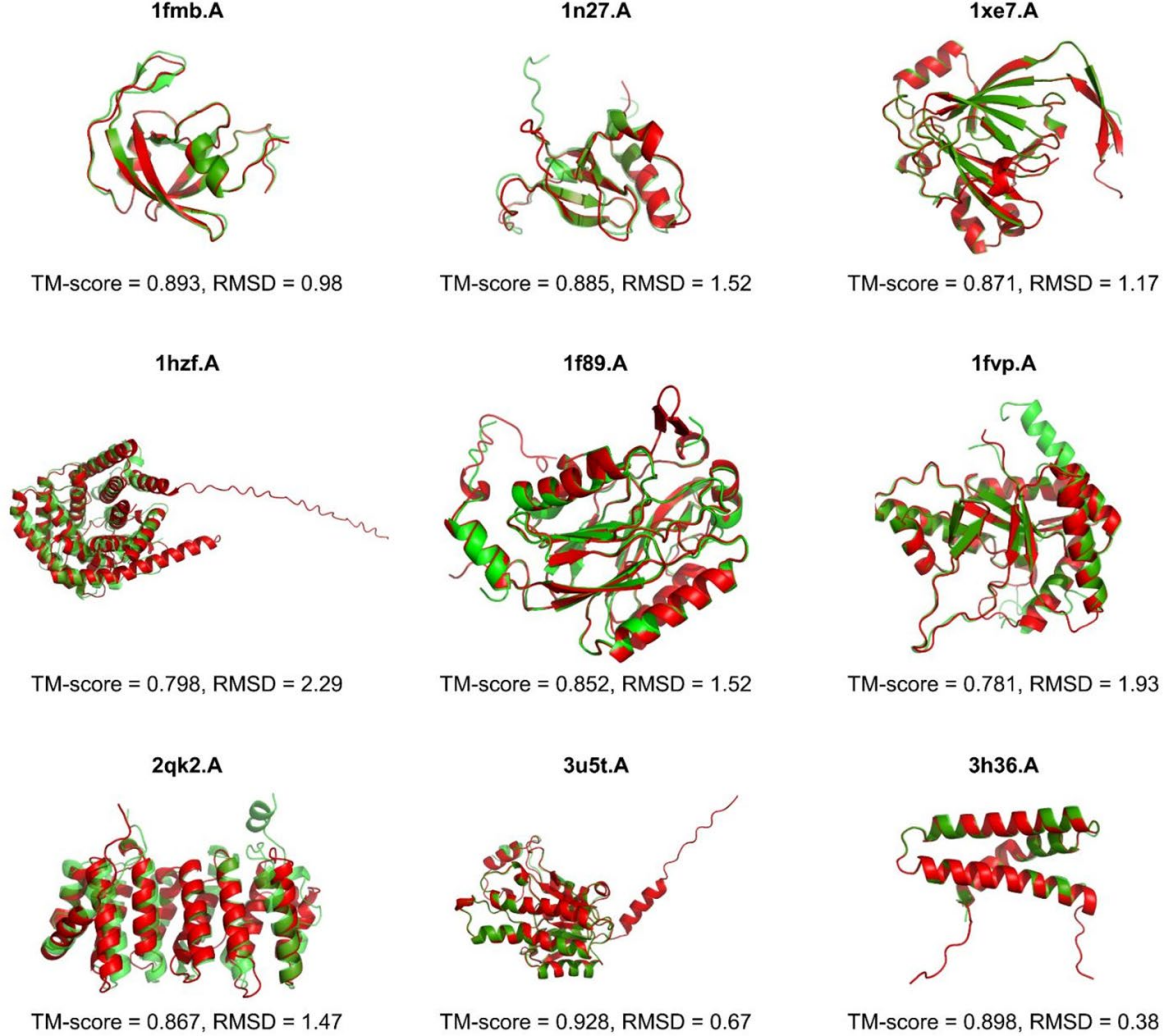
*De novo* protein designs by ProtSeqGen and their structural predictions. Native and predicted structures are shown in green and red, respectively

The sequences designed by ProtSeqGen were high consistent with their corresponding native sequence. Seven out of nine designed sequences achieved a recovery rate above 50% (Table 10). To further assess structural fidelity, we employed AlphaFold3 to predict the 3D structures of the designed sequences, which were compared against their respective native structures. As shown in Fig. 3, the native and predicted structures exhibit strong alignment, supported by high TM-scores and low RMSD values (average 1.3Å), confirming the high reliability of ProtSeqGen in sequence design.

**Table 10:** Performance evaluation of protein design methods in terms of structural accuracy. The **best** results are highlighted in bold.

Protein	Method	$\:\uparrow\:$ Recovery (%)	$\:\uparrow\:$ TM-score	$\:\downarrow\:$ RMSD (Å)	$\:\uparrow\:$ Stability score
1fmb.A	ProteinMPNN	42.69	0.324	4.23	8.071
	Pifold	**51.84**	0.862	1.62	**9.124**
	GeoSeqBuilder	50.38	0.831	1.97	8.012
	**ProtSeqGen**	50.96	**0.893**	**0.98**	8.398
1n27.A	ProteinMPNN	46.85	0.254	5.31	9.142
	Pifold	54.39	0.645	3.14	8.921
	GeoSeqBuilder	53.74	0.656	2.43	8.401
	**ProtSeqGen**	**55.21**	**0.885**	**1.52**	**9.328**
1xe7.A	ProteinMPNN	46.85	0.869	1.35	7.612
	Pifold	52.57	0.816	1.96	7.981
	GeoSeqBuilder	**54.92**	0.812	1.76	**9.441**
	**ProtSeqGen**	54.88	**0.871**	**1.17**	8.151
1hzf.A	ProteinMPNN	44.53	0.723	2.74	8.231
	Pifold	48.07	0.734	2.40	8.312
	GeoSeqBuilder	**49.52**	0.691	2.84	7.931
	**ProtSeqGen**	48.76	**0.798**	**2.29**	**8.956**
1f89.A	ProteinMPNN	47.26	0.694	1.64	**9.318**
	Pifold	**51.82**	0.825	2.54	7.048
	GeoSeqBuilder	50.72	**0.860**	1.54	8.276
	**ProtSeqGen**	51.65	0.852	**1.52**	7.887
1fvp.A	ProteinMPNN	44.59	0.825	2.56	9.091
	Pifold	**49.56**	0.824	1.80	8.801
	GeoSeqBuilder	48.53	**0.919**	**1.37**	8.062
	**ProtSeqGen**	47.87	0.781	1.93	**9.248**
2qk2.A	ProteinMPNN	48.32	0.826	1.56	8.401
	Pifold	53.26	0.834	2.79	8.512
	GeoSeqBuilder	52.68	0.827	1.85	8.871
	**ProtSeqGen**	**58.07**	**0.867**	**1.47**	**8.927**
3u5t.A	ProteinMPNN	45.13	0.791	2.39	8.721
	Pifold	53.74	0.753	2.06	8.556
	GeoSeqBuilder	53.86	0.765	2.12	9.351
	**ProtSeqGen**	**54.29**	**0.928**	**0.67**	**9.418**
3h36.A	ProteinMPNN	43.67	0.563	2.80	8.041
	Pifold	50.83	0.644	3.51	8.221
	GeoSeqBuilder	51.11	0.559	2.81	**8.951**
	**ProtSeqGen**	**55.13**	**0.898**	**0.38**	8.452

We also compared ProtSeqGen with three SOTA models (ProteinMPNN, PiFold, and GeoSeqBuilder) on the same set of proteins. As shown in Table 10, ProtSeqGen achieved significantly higher TM-score and lower RMSD values compared to all comparative methods. Notably, ProtSeqGen simultaneously achieved the best structural accuracy​ (TM-score and RMSD) for seven targets (1fmb.A, 1n27.A, 1xe7.A, 1hzf.A, 2qk2.A, 3u5t.A, and 3h36.A), outperforming​ the three competing methods. Moreover, we evaluated the folding stability of the nine designed proteins using the ‘Hybrid Score3’ proposed by Cho et al. [32]. Crucially, among these seven, the designs for 1n27.A, 1hzf.A, 2qk2.A, 3u5t.A ranked first in stability, while the remaining three (1fmb.A, 1xe7.A, and 3h36.A) secured second place.

In summary, these results demonstrate that ProtSeqGen robustly designs sequences that fold into native-like structures across diverse scaffolds, as validated by high structural accuracy and stability in AlphaFold3 predictions.

## Discussion

In summary, we developed ProtSeqGen, a graph-based deep learning framework for structure-guided protein sequence design that decodes central residue types with high accuracy. The superior performance of ProtSeqGen lies in its multi-scale geometric feature encoding framework. By fusing information through local geometric graphs​ and specifically leveraging backbone anchors​ and $\:C\beta\:$ geometry​ to strengthen the modeling of main-chain conformations, the framework enables more precise sequence design.

The inclusion of virtual atom nodes in ProtSeqGen serves to extend the protein backbone graph, introducing a global hub that aggregates and disseminates information across all residues. This design is inspired by the virtual node mechanism, which has been shown to enhance the performance of GNNs on various relational tasks [33, 34]. Critically, through systematic ablation studies, we identified that using two virtual nodes yields the optimal performance for our framework.

**Fig. 4:**
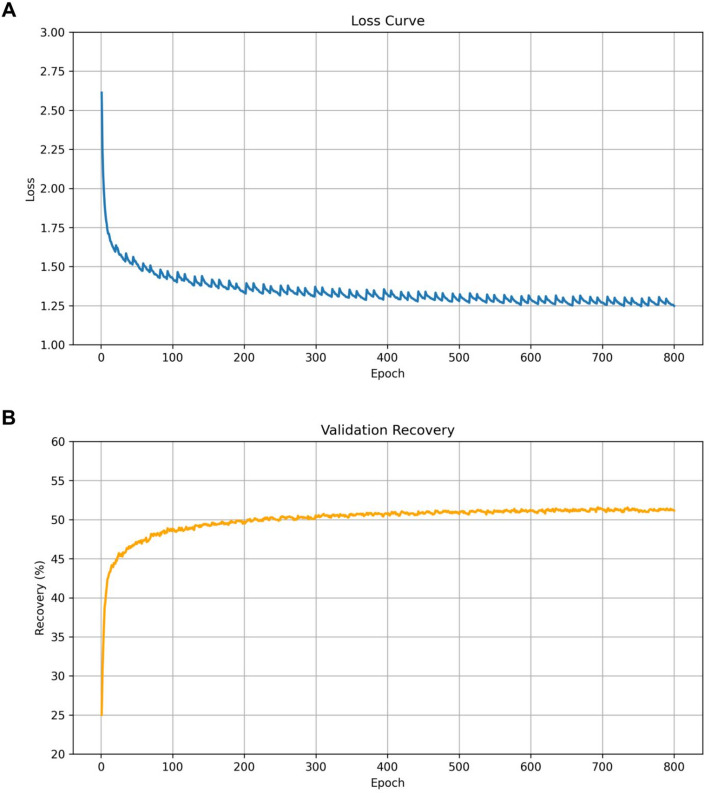
Convergence and performance assessment of ProtSeqGen on CATH 4.2. **A** The training loss curve; **B** The sequence recovery on validation set

ProtSeqGen was trained on the benchmarking dataset CATH 4.2, exhibiting highly reliable training dynamics as evidenced by the stable descent and convergence of the training loss (Fig. 4), which indicates a robust optimization process without overfitting. The pre-trained model was then rigorously validated on two independent testing sets, TS50 and TS500. Our results demonstrate that ProtSeqGen achieves both superior generalization performance and enhanced computational efficiency (Supplementary Table 7), relative to most existing SOTA approaches. When the pre-trained ProtSeqGen is applied to challenging benchmarks (IDRome-120 and TS45), it demonstrates robust sequence design capabilities on highly challenging targets (e.g., intrinsically disordered regions and free-modeling targets), significantly outperforming three representative methods (Supplementary Tables 8 and 9).

Furthermore, we explored whether further training (fine-tuning)​ the pre-trained ProtSeqGen model with additional relevant samples could enhance its performance on IDR and FM tasks, given the potentially low correlation between the CATH 4.2 training set and the IDRome-120 and TS45 benchmarks. Consequently, we developed a targeted fine-tuning strategy (Supplementary Text 2), and trained two distinct models: ProtSeqGen-FT-IDR for IDRome-120 and ProtSeqGen-FT-FM for TS45. As shown in Supplementary Table 10, both models show improved performance, with ProtSeqGen-FT-IDR​ exhibiting a notable recovery rate increase, likely attributable to the fine-tuning samples being derived from the same source dataset as IDRome-120. Our analysis suggests that ProtSeqGen excels in protein design tasks involving structural uncertainty or a lack of homologous templates, where traditional methods typically struggle.

Unlike traditional physics-based methods, ProtSeqGen requires no expert-driven customization for specific design tasks, making protein design more broadly accessible. When provided with various *de novo* backbones as input, ProtSeqGen successfully generates amino acid sequences that fold accurately into their corresponding target tertiary structures, as evidenced by high sequence recovery, high TM-scores, and low RMSD values (Supplementary Figs. 1–3). Crucially, ProtSeqGen-generated sequences exhibit superior stability over existing models, indicating high practical application potential.

Despite these strengths, ProtSeqGen presents several areas for further improvement. First, while effective for designing ​a diverse set of protein scaffolds (all-$\:\alpha\:$, all-$\:\beta\:$, $\:\alpha\:/\beta\:$, $\:\alpha\:+\beta\:$ folds, membrane proteins, and TIM-barrels)​, its transferability to other protein classes remains to be fully established. Second, the current study is limited by ​the lack of high-throughput structural validation​ of the generated protein designs. Lastly, training strategies tailored to distinct protein classes remain a crucial and open area for further investigation.

## Conclusion

In this study, we developed ProtSeqGen, a graph-based deep learning framework for structure-guided protein sequence design. Leveraging graph neural networks and a novel virtual node mechanism, the model integrates multi-scale structural and contextual information, achieving high accuracy in decoding residue types. Comprehensive benchmarking across CATH 4.2, TS50, TS500, IDRome-120, and TS45 reveals that ProtSeqGen attains exceptional performance in sequence generation and exhibits strong generalization across diverse protein folds, highlighting its practical applicability. While effective, specific areas, such as experimental validation of designed proteins, merit deep exploration. In summary, ProtSeqGen provides a robust and accessible tool for computational protein design, with the potential to accelerate the creation of novel proteins for therapeutic and biotechnological applications.

## Methods

### Dataset collection and preprocessing

To assess model generalization across distinct protein folds, we use the CATH 4.2 dataset curated by Ingraham et al. [35]. This dataset comprises full chains up to length 500, and structures have been partitioned with 40% non-redundancy by their CATH topology classification. The resulting training, validation, and test sets contain 18,204, 608, and 1120 structures, respectively [19, 22, 36]. To present a comprehensive comparison, we further evaluate our pre-trained model on two smaller benchmarks, TS50 and TS500, which comprise 50 and 500 proteins with native structures, respectively [14].

Moreover, we assess the capability of the model on specialized datasets featuring intrinsically disordered regions (IDRs) [26] and free-modeling (FM) targets [29]. First, we compiled a IDRoom​ dataset (named as ‘IDRoom-120’) by manually curating 120 proteins with diverse sequence lengths, from an initial pool of 1, 508 annotated candidates (Supplementary Text 1). Second, we employed TS45 (from CASP15), a highly challenging benchmark for *de novo* protein design consisting of FM targets with no homology to known structures [30]. Detailed information of these datasets is summarized in Table 1.

### ProtSeqGen modeling

#### Generation of the local geometric graph

In order to capture the local conformational features, a protein backbone is partitioned into local fragments, each defined as a structural unit comprising two consecutive residues [37]. Corresponding to each fragment, we construct a residue-level geometric graph, which serves as the structural foundation for subsequent feature extraction.

#### Feature extraction at residue scale

##### Definition of local orthogonal basis

For any residue $\:i$, a local orthogonal basis $\:{Q}_{i}\:$is defined using the global coordinates of its backbone atoms: the nitrogen ($\:{N}_{\boldsymbol{i}}$), the alpha carbon ($\:{{C}_{\alpha\:}}_{i}$), and the carbonyl carbon ($\:{C}_{\boldsymbol{i}}$), where $\:{N}_{\boldsymbol{i}},\:{{C}_{\alpha\:}}_{i},{C}_{\boldsymbol{i}}\in\:{R}^{3}$ [23]. First, we construct three vectors from these coordinates:

1 $$\:b={{C}_{\alpha\:}}_{i}-{N}_{\boldsymbol{i}},c={C}_{\boldsymbol{i}}-{{C}_{\alpha\:}}_{i},a=b\times\:c$$

Their corresponding unit vectors are:

2 $$\:\overrightarrow{b}=\frac{b}{\parallel\:b\parallel\:}\text{},\overrightarrow{a}=\frac{a}{\parallel\:a\parallel\:}\text{},\overrightarrow{c}=\widehat{b}\times\:\widehat{a}$$

The resulting basis $\:{Q}_{i}=\left[\overrightarrow{b},\overrightarrow{a},\overrightarrow{c}\right]$ is then used to project atomic coordinates and compute local geometric features at the ‘node’ and ‘edge’ levels.

##### Generating the intra-residue features

To accurately represent the local structural environment of each residue, we extract intra-residue features from its backbone atoms ($\:N,{C}_{\alpha\:},C,O$) and the $\:C\beta\:$ atom. The $\:C\beta\:$ atom plays a critical role in determining side-chain conformation. To better capture residue-level geometry, we include its coordinates as a key local feature. These coordinates are computed from the local reference frame defined by the $\:N$, $\:{C}_{\alpha\:}$, and $\:C$ atoms using standard peptide geometry [21, 38, 39]. For glycine, which lacks a $\:C\beta\:$ atom, a virtual $\:C\beta\:$ position is generated using the same method to ensure feature consistency across all residue types. The calculation of $\:C\beta\:$ geometry is as follows:

3 $$\:{C\beta\:}_{i}\:=-0.58273431\overrightarrow{a}+0.56802827\overrightarrow{b}-0.54067466\overrightarrow{c}+{{C}_{\alpha\:}}_{i}$$

To better capture the geometric relationships among atoms within a residue, we introduce atom-level virtual nodes. The positions of these nodes are parameterized​ within the local residue frame. They serve as abstract anchors that can enhance the representational capacity of graph neural networks, as demonstrated in prior works on attention mechanisms [33, 34].

The position of the *g*-th virtual node is computed as:

4 $$ vn_{g} = v_{x} \cdot \vec{a} + v_{y} \cdot \vec{b} + v_{z} \cdot \vec{c} + \vec{r} $$

where $\:\overrightarrow{r}$ is the position vector of the $\:{C}_{\alpha\:}$ atom.

For the residue $\:i$, we compute the pairwise distances between all its backbone atoms ($\:{N}_{i},{{C}_{\alpha\:}}_{i},{C}_{\boldsymbol{i}},{O}_{\boldsymbol{i}}$), the $\:{C\beta\:}_{i}$ atom, and the virtual atoms ($\:{vn}_{i,1}$,…$\:{vn}_{i,p},\dots\:{vn}_{i,q},\dots\:$) within the local coordinate frame. These distances are encoded using a radial basis function (RBF) kernel to obtain the distance feature vector $\:{D}_{i}^{V}$​:

5 $$ D_{i}^{V} = \left[ {RBF\left( {\left\| {N_{i} - C_{{\alpha i}} } \right\|} \right), \ldots ,RBF\left( {\left\| {C\alpha _{i} - O_{i} } \right\|} \right), \ldots ,RBF\left( {\left\| {vn_{{i,p}} - vn_{{i,q}} } \right\|} \right)} \right] $$

The angle feature vector $\:{A}^{V}$ is derived from both dihedral angles and local bond angles. We define three dihedral angles ($\:\varphi\:,\psi\:,\omega\:$) and three bond angles ($\:\alpha\:,\:\beta\:,\:\gamma\:$). The dihedral angle defined by four consecutive atoms $\:E,F,G,H$ (representing two intersecting plants ($\:E,F,G$) and ($\:F,G,H$)) is calculated as:

6 $$\:f(E,F,G,H)=arctan2\left(\left({\overrightarrow{n}}_{2}\times\:{\overrightarrow{n}}_{1}\right)\cdot\:{\overrightarrow{s}}_{2},\:\:\:{\overrightarrow{n}}_{1}\cdot\:{\overrightarrow{n}}_{2})\cdot\:\:\parallel\:{\overrightarrow{s}}_{2}\parallel\:\right)$$

where

$$\:{\overrightarrow{s}}_{1}=F-E,{\overrightarrow{s}}_{2}=G-F,{\overrightarrow{s}}_{3}=H-G$$

$$\:{n}_{1}={\overrightarrow{s}}_{1}\times\:{\overrightarrow{s}}_{2}\:\:,\:{n}_{2}={\overrightarrow{s}}_{2}\times\:{\overrightarrow{s}}_{3}$$

Specifically,

$$\:\varphi\:=f\left({C}_{\boldsymbol{i}-1},{N}_{i},{{C}_{\alpha\:}}_{i},{C}_{\boldsymbol{i}}\right),\:\psi\:=f\left({N}_{i},{{C}_{\alpha\:}}_{i},{C}_{\boldsymbol{i}},{N}_{i+1}\right),\:\:\omega\:=f\:({{C}_{\alpha\:}}_{i},{C}_{\boldsymbol{i}},{N}_{i+1},{{C}_{\alpha\:}}_{i+1}).$$

The local bond angle formed by vector $\:\overrightarrow{EF}$ and $\:\overrightarrow{FG}$ is given by:

7 $$\:l(E,F,G)=arccos\left({\overrightarrow{u}}_{0}\cdot\:{\overrightarrow{u}}_{1}\right)$$

where

$$\:{\overrightarrow{u}}_{0}=\:\frac{E\:-\:F}{\parallel\:E\:-\:F\parallel\:},\:{\overrightarrow{u}}_{1}\:=\:\frac{G\:-\:F}{\parallel\:G\:-\:F\parallel\:}$$

These yields the specific bond angles:

$$\:\alpha\:=l({N}_{i},{{C}_{\alpha\:}}_{i},{C}_{\boldsymbol{i}}),\:\beta\:=l({{C}_{\alpha\:}}_{i},{C}_{\boldsymbol{i}},{N}_{i+1}),\:\gamma\:=l({C}_{\boldsymbol{i}},{N}_{i+1},{{C}_{\alpha\:}}_{i+1})$$

Taken above together, the angle feature vector $\:{A}_{i}^{V}$ is constructed as the sine and cosine encodings of all six angles:

8 $$\:{A}_{i}^{V}=[\mathrm{cos}\left(\varphi\:\right),\mathrm{sin}\left(\varphi\:\right),\dots\:,\mathrm{cos}\left(\gamma\:\right),\:\mathrm{s}\mathrm{i}\mathrm{n}(\gamma\:\left)\right]$$

For each internal backbone atom $\:t\in\:(N,C,O)$ in residue $\:i$, we calculate its vector relative to $\:{{C}_{\alpha\:}}_{i}$ and project it onto the local orthonormal basis $\:[\overrightarrow{a},\overrightarrow{b},\overrightarrow{c}]$ (defined in Eq. 2). The resulting coordinates are normalized to obtain a unit direction vector. The concatenation of these unit vectors forms the direction feature $\:{Q}_{i}^{V}$:

9 $$ Q_{i}^{V} = \left[ {\frac{{v_{N} }}{{\left\| {v_{N} } \right\|}},\frac{{v_{C} }}{{\left\| {v_{C} } \right\|}},\frac{{v_{O} }}{{\left\| {v_{O} } \right\|}}} \right] $$

where $$ v_{t} = \left( {t_{i} - C_{{\alpha i}} } \right) \cdot (\vec{a},\vec{b},\vec{c}) $$.

##### Generating the inter-residue features

In this subsection, we further calculate the pairwise features between residues, namely the distance feature $\:{D}^{E}$, the angle feature $\:{A}^{E}$, and direction feature $\:{Q}^{E}$. Given a residue pair $\:(i,\:j)$, the distance feature $\:{D}_{ij}^{E}$ is computed via RBF kernel applied to the distances between their constituent atoms:

10 $$ D_{{ij}}^{E} = \left[ {RBF\left( {\left\| {m_{i} - m_{j} } \right\|} \right),...,RBF\left( {\left\| {vn_{i} - vn_{j} } \right\|} \right)} \right] $$

where $\:{m}_{i}\in\:({N}_{i},{{C}_{\alpha\:}}_{i},{C}_{\boldsymbol{i}},{O}_{\boldsymbol{i}},{C\beta\:}_{i})$, and $\:{m}_{j}\in\:({N}_{j},{{C}_{\alpha\:}}_{j},{C}_{\boldsymbol{j}},{O}_{\boldsymbol{j}},{C\beta\:}_{j})$. The virtual nodes $\:{vn}_{i}$ and $\:{vn}_{j}$ are from residue $\:i$ and $\:j$, respectively.

The angle feature $\:{A}^{E}$ encodes the relative 3D orientation between the local frames of residues $\:i$ and $\:j$. It is derived from the rotation matrix $\:{R}_{ij}$ reflecting how the local coordinate system of residue $\:i$ must be rotated to align with that of residue $\:j$:

$$\:{R}_{ij}={Q}_{i}^{T}{Q}_{j}$$

We then convert $\:{R}_{ij}$ into a unit quaternion representation $\:{A}^{E}=\left(w,x,y,z\right)$ using the trace-based method:

11 $$ w = 0.5\sqrt {1 + Tr\left( {R_{{ij}} } \right)} ,x = \frac{{R_{{ij}} \left( {3,2} \right) - R_{{ij}} \left( {2,3} \right)}}{{4w}},y = \frac{{R_{{ij}} \left( {1,3} \right) - R_{{ij}} \left( {3,1} \right)}}{{4w}},z = \frac{{R_{{ij}} \left( {2,1} \right) - R_{{ij}} \left( {1,2} \right)}}{{4w}} $$

The direction feature $\:{Q}_{ij}^{E}$ represents the relative orientation of each backbone atom $\:{m}_{j}$ in residue $\:j$ with respect to the $\:{{C}_{\alpha\:}}_{i}$ atom of residue $\:i$. For an atom $\:{m}_{j}\in\:\{{N}_{j},\:{{C}_{\alpha\:}}_{i},\:{C}_{j},\:{O}_{j}\}$, we compute its vector relative to $\:{{C}_{\alpha\:}}_{i}$, project it onto the local frame $\:[\overrightarrow{a},\overrightarrow{b},\overrightarrow{c}]$ of residue $\:i$, and normalize:

12 $$ Q_{{ij}}^{E} = \left[ {\frac{{e_{N} }}{{\left\| {e_{N} } \right\|}},\frac{{e_{{c\alpha }} }}{{\left\| {e_{{c\alpha }} } \right\|}},\frac{{e_{C} }}{{\left\| {e_{C} } \right\|}},\frac{{e_{O} }}{{\left\| {e_{o} } \right\|}}} \right] $$

where $$ e_{m} = \left( {m_{j} - C_{{\alpha i}} } \right) \cdot (\vec{a},\vec{b},\vec{c}) $$.

#### Comprehensive residue representation via feature fusion

For each local geometric graph, the residue-level node features (distance $\:{D}^{V}$, angle $\:{A}^{V}$, and orientation $\:{Q}^{V}$) and the inter-residue edge features (pairwise distance $\:{D}^{E}$, angle $\:{A}^{E}$, and direction $\:{Q}^{E}$) are separately encoded by two independent feedforward neural networks (FNN) [40]. Each FNN integrates its three input feature matrices and projects them into a unified ​outputs​ a unified 128-dimensional embedding. As illustrated in Fig. 1, the resulting intra- and inter-residue embeddings, denoted $\:{F}^{V}$ and $\:{F}^{E}$ are computed as follows:

13 $$\:{F}^{V}=\sigma\:\left({W}_{2}^{V}*\phi\:\right({W}_{1}^{V}[{D}^{V},{A}^{V},{Q}^{V}]+{b}_{1}^{V})+{b}_{2}^{V})\:$$

14 $$\:{F}^{E}=\sigma\:\left({W}_{2}^{E}*\phi\:\right({W}_{1}^{E}[{D}^{E},{A}^{E},{Q}^{E}]+{b}_{1}^{E})+{b}_{2}^{E})$$

where $\:{W}_{\mathrm{1,2}}^{E}$, $\:{W}_{\mathrm{1,2}}^{V}$ are learnable weight matrices, $\:{b}_{\mathrm{1,2}}^{E}$, $\:{b}_{\mathrm{1,2}}^{V}$ are the corresponding bias terms, and both $\:\sigma\:(\cdot\:)$ and $\:\phi\:(\cdot\:)$ denote the ReLU activation function. The original feature dimensions are $\:{D}^{V},{A}^{V},{Q}^{V}\in\:{R}^{101}\:$and $\:{D}^{E},{A}^{E},{Q}^{E}\in\:{R}^{412}$.

The fused feature maps $\:{F}^{V}$ and $\:{F}^{E}$ are subsequently fed into a Message Passing Neural Network (MPNN). The MPNN adaptively integrates​ residue-intrinsic properties and spatial relationships through iterative message passing, yielding enhanced residue representations for the final sequence decoding.

#### Feature refinement through the MPNN network

Given an input graph $\:G=(V,E)$, each node $\:{v}_{i}\in\:V$ represents $\:i$-th residue and is connected to its neighboring residue $\:{v}_{j}$ via an edge $\:{e}_{ij}\in\:E$​. The feature refinement process in a $\:N$-layer MPNN proceeds as follows.

First, the node and edge embeddings are initialized from the outputs of the FNN encoders and projected to a consistent dimensionality using Layer normalization:

15 $$\:{h}_{i}^{\left(0\right)\text{}}\leftarrow\:LayerNorm\left({W}_{h}{F}^{V}+{b}_{h}\right)$$

16 $$\:{\:e}_{ij}^{\left(0\right)}\leftarrow\:LayerNorm\left({W}_{e}{F}^{E}+{b}_{e}\right)$$

Here, $\:{h}_{i}^{\left(0\right)\text{}}$​ encodes residue-specific features (distances, angles, orientations), while $\:{\:e}_{ij}^{\left(0\right)}$encodes pairwise geometric relations.

At each layer $\:k$ of the MPNN, a message for residue $\:i$ is computed ​by aggregating information from its neighbor $\:j\in\:N\left(i\right)$:

17 $$\:{m}_{i}^{\left(k\right)}=\sum\:_{j\in\:N\left(i\right)}{\phi\:}_{m}\left({h}_{i}^{\left(k-1\right)},{h}_{j}^{\left(k-1\right)},{e}_{ij}^{\left(k-1\right)}\right)$$

where $\:\phi\:(\cdot\:)$ is a two-layer FFN with a ReLU function. The node embedding is subsequently updated as:

18 $$\:{h}_{i}^{\left(k\right)}=LayerNorm\left({h}_{i}^{\left(k-1\right)}+Dropout\left({\phi\:}_{u}\boldsymbol{}\left({h}_{i}^{\left(k-1\right)}\boldsymbol{},{m}_{i}^{\left(k-1\right)}\boldsymbol{}\right)\right)\right)\:$$

Furthermore, a scaled dot-product attention mechanism is incorporated between MPNN layers to capture long-range dependencies. The queries ($\:U$), keys ($\:K$), and values ($\:V$) are projected from the node embeddings:

19 $$\:{U}_{i}={W}_{U}{h}_{i}^{\left(k\right)},{K}_{i}={W}_{K}{h}_{i}^{\left(k\right)},{V}_{i}={W}_{V}{h}_{i}^{\left(k\right)}$$

The attention weights integrate geometric biases derived from the edge embeddings $e_{{ij}}^{{\left( k \right)}} :$​

20 $$\:{\alpha\:}_{ij}=\frac{exp\left({U}_{i}^{\top\:}{K}_{j}/\sqrt{{d}_{\mathrm{n}}}+b\left({e}_{ij}^{\left(k\right)}\right)\right)}{\sum\:_{j\in\:{NS}_{i}}exp\left({U}_{i}^{\top\:}{K}_{j}/\sqrt{{d}_{\mathrm{n}}}+b\left({e}_{ij}^{\left(k\right)}\right)\right)}$$

where $b\left( \cdot \right)$ denotes a learnable linear function that maps the edge embedding to a scalar bias. The attended features are then fused with a residual connection:

21 $$\:{h}_{i}^{\left(k\right)}=LayerNorm\left({h}_{i}^{\left(k\right)}+{W}_{O}\sum\:_{j\in\:{NS}_{i}}{\alpha\:}_{ij}{v}_{j}\right)$$

Finally, the steps defined by Eqs. 17–21 are repeated for $\:N$ layers. Each residue embedding progressively integrates local and non-local contextual information, yielding refined features for downstream sequence design.

#### Residue type prediction with MLP classifier

As described in the above section, the refined residue embedding $\:{h}_{i}^{\left(N\right)}$ is fed into an MLP classifier for residue type prediction (see Fig. 1). The classifier outputs a vector of logits $\:{{y}_{i}\in\:\mathrm{R}}^{20}$, corresponding to the 20 standard amino acid types:

22 $$\:{y}_{i}=MLP\left({h}_{i}^{\left(N\right)}\right)$$

The predicted residue type $\:{\widehat{Y}}_{i}$ ​is obtained by applying the softmax function to the logits and selecting the class with maximal probability:

23 $$\:{\widehat{Y}}_{i}=\underset{l=\{1,\dots\:,20\}}{\mathrm{argmax}}\left({\sigma\:}_{l}\right({y}_{i}\left)\right),\:\mathrm{w}\mathrm{h}\mathrm{e}\mathrm{r}\mathrm{e}\:{\sigma\:}_{l}\left({y}_{i}\right)=\frac{{e}_{{y}_{i,l}}}{\sum\:_{j=1}^{20}{e}_{{y}_{i,j}}}$$

### ProtSeqGen model training and calibration

ProtSeqGen is optimized via Adam optimizer with weight decay and label smoothing to bolster generation. A Noam scheduler with linear warmup adjusts the learning rate to stabilize training and ensure robust convergence [41]. Training leverages automatic mixed precision and gradient clipping, with a fixed seed guaranteeing reproducibility. Hyperparameters listed in Supplementary Table 1 are determined through manual tuning. Prediction results are reported as the mean and standard deviation over 10 independent training runs.

### Evaluation metrics

In this study, we employ six metrics to evaluate the performance of ProtSeqGen from three perspectives. At the sequence level, sequence recovery [42] and perplexity [43] are used to evaluate the difference between the designed and native sequences. At the structure level, RMSD (Root Mean Square Deviation) [22], TM-score (Template Modeling Score) [23], and Stability score (‘Hybrid Score3’ proposed by Cho et al.) [32] are used to evaluate the difference between the 3D structure of the designed sequence and the target structure. In addition, Loss reflects the difference between the predicted and real value after modeling training. The definition of these metrics is presented as follows:

24 $$\:Recovery=\frac{1}{N}\sum\:_{i=1}^{N}f({s}_{i}^{d}={s}_{i}^{n})$$

25 $$ Perplexity = {\mathrm{exp}}\left( { - \frac{1}{N}\sum\nolimits_{{i = 1}}^{N} {logP(s_{i} |s_{1} ,s_{2} , \ldots ,s_{{i - 1}} )} } \right) $$

26 $$ RMSD = \sqrt {\frac{1}{N}\sum\nolimits_{{i = 1}}^{N} {\left\| {r_{i}^{d} - r_{i}^{n} } \right\|} ^{2} } $$

27 $$\:TM-score=\mathrm{max}\left(\frac{1}{{L}_{n}}\sum\:_{i=1}^{{L}_{a}}\frac{1}{1+{\left({d}_{i}/\left({d}_{0}\right({L}_{n}\left)\right)\right)}^{2}}\right)$$

28 $$\:Loss=-\frac{1}{N}\sum\:_{i=1}^{N}\sum\:_{a=1}^{20}{y}_{i,a}\mathrm{l}\mathrm{o}\mathrm{g}\left({p}_{i,a}\right)$$

where $\:N$ is the length of protein sequence $\:S=({s}_{1},{s}_{2},\dots\:,{s}_{N})$. $\:{s}_{i}^{d}$ and $\:{s}_{i}^{n}$ denote $\:i-th$ residue. Function $\:f\left(*\right)$ is an indicator with Boolean values. $\:P\left({s}_{i}\right|{s}_{1},{s}_{2},\dots\:,{s}_{i-1})$ is the probability of the correct residue $\:{s}_{i}$ based on all previous residues. $\:{r}_{i}^{d}$ and $\:{r}_{i}^{n}$ represent the 3D coordinates of $\:i-th$ atom in the designed and native structures, respectively. $\:{L}_{n}$ and $\:{L}_{a}$ are defined as the length of the native sequence and the number of aligned residues after alignment. $\:{d}_{i}$ denotes the distance between the $\:i-th$ pair of aligned residues. Scale factor $\:{d}_{0}\left({L}_{n}\right)=1.24\sqrt{{L}_{n}-1.5}-1.8$. The one-hot vector $\:{y}_{i,a}$ represents $\:i-th$ real residue. $\:{p}_{i,a}$ is the probability that the $\:i-th$ residue is predicted as ‘a’.

### Comparisons with existing methods

To ensure a comprehensive evaluation, we compared ProtSeqGen against fifteen representative methods spanning three primary categories: multilayer perceptron-based techniques such as SPIN [14], SPIN2 [44], and Wang’s model [45]; convolutional neural network-based approaches including SPROF [18], ProDCoNN [17], and DenseCPD [46]; as well as graph-based models, namely StructGNN [19], GraphTrans [35], GVP [36], GCA, AlphaDesign [25], ProteinMPNN [21], Frame2seq [47], PiFold [22], and GeoSeqBuild [23]. All models were trained on CATH 4.2 and subsequently evaluated on the independent testing sets TS50, TS500, IDRome-120, and TS45 (Table 1). The comparison study employed identical evaluation metrics.

### Ablation study

The ablation study is designed to prove the validity of the proposed ProtSeqGen framework from three perspectives. First, structural configuration analysis is conducted to determine the optimal combination of $\:C\beta\:$ geometry and virtual nodes. Second, we sequentially removed (1) intra-residue features, (2) inter-residue features, or (3) attention layer in MPNN, generating five ablated variants ($\:{M}_{1}$-$\:{M}_{5}$) for comparison with the complete ProtSeqGen framework (Table 3). Finally, we performed a leave-one-out analysis on all six distinct feature types ($\:{D}^{V}$, $\:{A}^{V}$, $\:{Q}^{V}$, $\:{D}^{E}$, $\:{A}^{E}$, and $\:{Q}^{E}$), to pinpoint their individual importance (Fig. 1).

### Simulation environment

The codes of ProtSeqGen were developed and debugged by using PyTorch 2.4.1 and Python 3.10.10 under the environment with 12th Gen Intel^®^ Core™ i9-12900 K processor and 128 GB RAM. All the simulations were performed with CUDA 12.1.105 on NVIDIA GeForce RTX A4000 32G graphics card. The 3D structure predicted by AlphaFold 3 was generated using the AlphaFold Server (https://alphafoldserver.com/).

## Electronic Supplementary Material

Below is the link to the electronic supplementary material.

Supplementary Material 1

## Data Availability

The data that support the findings of this study are derived from the following public domain resources: the CATH database (version 4.2, http://www.cathdb.info) [48], the standard benchmarks (TS50/TS500) [14], IDRome [26], and CASP15 [30]. The source code of ProtSeqGen is freely available on our Github repository https://github.com/NJAU-CDSIC/ProtSeqGen. The code of all SOTA models is also provided. Moreover, the benchmarking datasets (CATH 4.2, TS50, TS500, IDRome-120, and TS45) used in this study are publicly accessible and can be downloaded from our Github.
