# Supplementary material for: ProtSeqGen: a novel deep learning model for protein sequence design
Source: BMC Bioinformatics. 2026 May 26;27:156. doi: 10.1186/s12859-026-06482-4 (PMC13390367; doi:10.1186/s12859-026-06482-4)
Supplement: Supplementary file 1 — Supplementary Material 1 [file 12859_2026_6482_MOESM1_ESM.docx]

**Supplementary Materials**

**ProtSeqGen: a novel deep learning model for protein sequence design**

Qiang Gao^1,2^, Zhijin Li^3^, Yang Deng^4^, Zhiwei Ji^1,2,^ *

^1^College of Artificial Intelligence, Nanjing Agricultural University, No. 666 Binjiang Avenue, Nanjing, Jiangsu 211800, China

^2^Center for Data Science and Intelligent Computing, Nanjing Agricultural University, No. 666 Binjiang Avenue, Nanjing, Jiangsu 211800, China

^3^Department of Neurosurgery, The First Affiliated Hospital of USTC (Anhui Provincial Hospital), Division of Life Science and Medicine, University of Science and Technology of China, Hefei, Anhui 230036, China

^4^School of Life Science and Technology, Harbin Institute of Technology, Harbin, Heilongjiang 150001, China

*Corresponding author: Zhiwei Ji ([Zhiwei.Ji@njau.edu.cn](mailto:Zhiwei.Ji@njau.edu.cn))

**Supplementary Text**

**Supplementary Text 1.** IDRome-120 dataset curation pipeline

In this study, we generated a novel stratified dataset, IDRome-120, by refining the raw IDRome entries. The construction procedure is detailed as follows:

**1) Data source**

Our pipeline commenced with the raw IDRome dataset, consisting of 28, 058 entries.

**2) Deduplication**

We eliminated sequence redundancy using a 90% sequence identity clustering criterion, reducing the dataset to 27, 223 non-redundant sequences to prevent model overfitting to repetitive patterns.

**3) Structure alignment and cleaning**

Utilizing UniProt IDs and residue ranges, we retrieved corresponding 3D structures from the PDB/CIF repositories. Structures were standardized into the {UniProt}_{start}_{end}.pdb format, and only backbone atoms ($N$, $C_{\alpha}$, $C$, $O$) were extracted into a JSONL file. A stringent validation step ensured that the filename-defined length matched the actual coordinate length, removing any entries with inconsistencies or parsing failures (remaining: 1, 553 samples).

**4) Quality control**

A final quality control step was conducted to exclude structures with invalid or missing backbone coordinates (notably NaN values). After filtering out 45 defective samples lacking valid $C_{\alpha}$ atoms, we finalized a high-fidelity dataset of 1, 508 samples for rigorous model benchmarking.

**5) Dataset curation for model testing**

All 1, 508 sequences are shorter than 200 residues. We randomly curated a subset of 120 sequences, named IDRome-120, from this pool, stratifying them equally into four length intervals (0-50, 51-100, 101-150, and 151-200 residues), with 30 sequences per interval (**Supplementary Table 6**).

**Supplementary Text 2.** Fine-tunning of ProtSeqGen for IDRome-120 and TS45

In this study, we collected relevant samples for fine-tuning ProtSeqGen and derived two specialized models: ProtSeqGen-FT-IDR for IDRome-120 and ProtSeqGen-FT-FM for TS45. The data collection protocol is as follows.

**1) Training data for ProtSeqGen-FT-IDR**

As detailed in the Methods section, after removing the 120 samples used to construct IDRome-120 from the initial pool of 1, 508 annotated proteins, 1, 388 samples remained. These were randomly divided into a training set of 1, 250 samples and a validation set of 138 samples. Using these 1, 388 samples, we further fine-tuned the ProtSeqGen model (pre-trained on the CATH 4.2 dataset) to obtain ProtSeqGen-FT-IDR.

**2) Training data for ProtSeqGen-FT-FM**

Since TS45 is constructed from the FM targets in CASP15, we collected 500 non-redundant FM samples from historical CASP events, including 165 from CASP8, 128 from CASP10, 105 from CASP11, 55 from CASP12, 32 from CASP13, and 15 from CASP14. All these samples were randomly divided into two groups: 450 for training, and 45 for validation. Similarly, we obtained ProtSeqGen-FT-FM.

**Supplementary Figures**


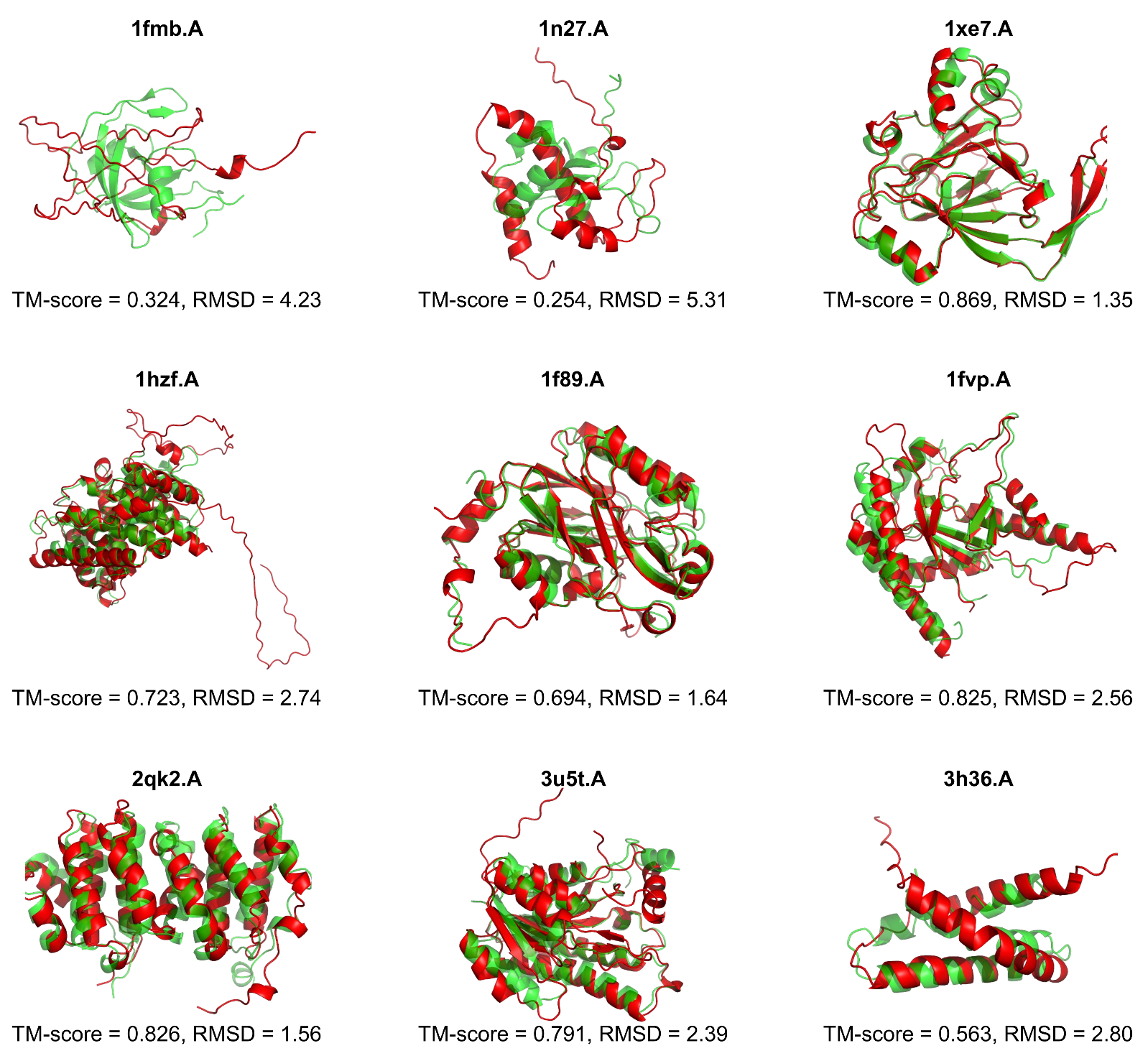


**Supplementary Figure 1.** *De novo* protein designs by ProteinMPNN and their structural predictions. Native and predicted structures are shown in green and red, respectively.


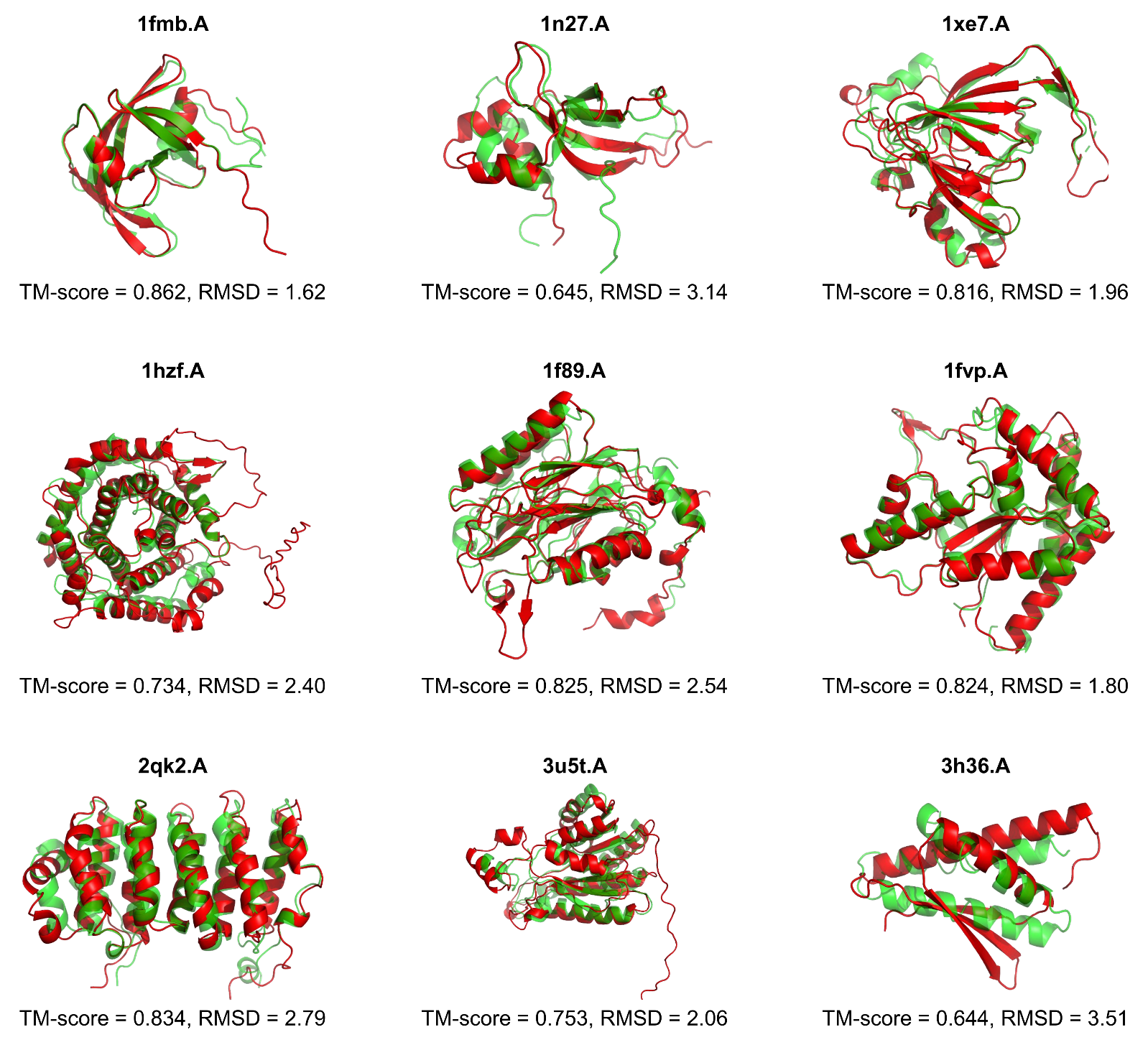


**Supplementary Figure 2.** *De novo* protein designs by PiFold and their structural predictions. Native and predicted structures are shown in green and red, respectively.


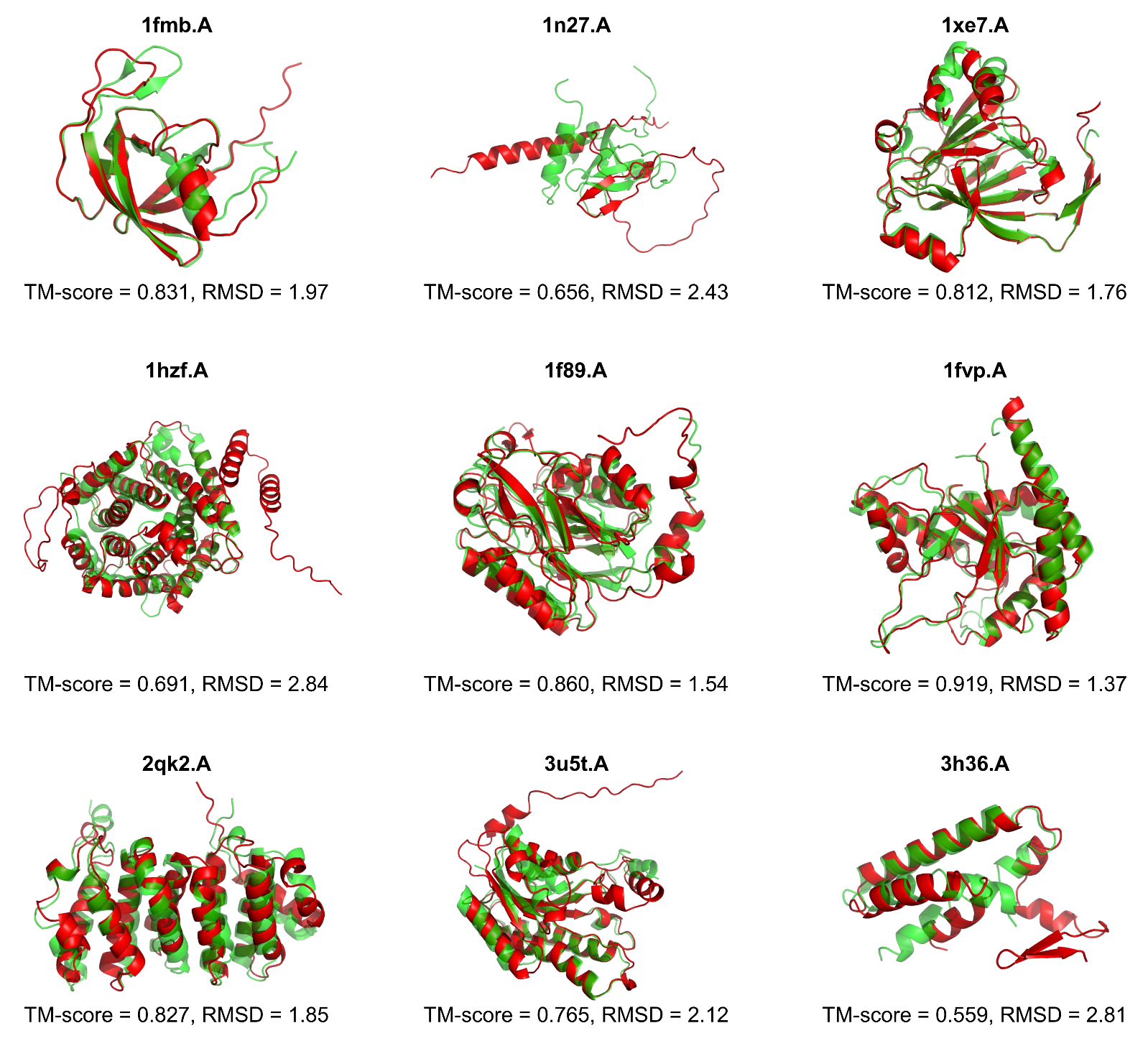


**Supplementary Figure 3.** *De novo* protein designs by GeoSeqBuilder and their structural predictions. Native and predicted structures are shown in green and red, respectively.

**Supplementary Tables**

**Supplementary Table 1.** The Hyperparameters of ProtSeqGen

| **Parameters** | **Value** |
| --- | --- |
| Max_protein_length | 10000 |
| Node_features | 128 |
| Edge_features | 128 |
| Optimal_virtual_atoms | 2 |
| MPNN_hidden_dim | 128 |
| MPNN_k_neighbors | 48 |
| MPNN_augment_eps | 0.05 |
| MPNN_attention layer | 1 |
| MPNN_dropout | 0.2 |
| Optimizer | Adam |
| Batch size | 1800 |
| Epoch | 800 |
| Warmup steps | 4000 |
| Label smoothing weight | 0.1 |
| Gradient clipping | 0.8 |

**Supplementary Table 2.** Statistical comparison on TS50 between ProtSeqGen and other methods (mean $\pm$ 95%CI)

| **Comparison** | **Metrics** | **Mean difference** | **95% CI** | **p-value** |
| --- | --- | --- | --- | --- |
| ProtSeqGen vs. ProteinMPNN | Recovery (%) $\boldsymbol{\uparrow}$ | 4.1505 | (4.0404, 4.2607) | **7.72E-14** |
|  | Perplexity $\boldsymbol{\downarrow}$ | -0.1152 | (-0.1669, -0.0636) | **1.21E-05** |
|  | Loss $\boldsymbol{\downarrow}$ | -0.1054 | (-0.1191, -0.0917) | **1.06E-07** |
| ProtSeqGen vs. PiFold | Recovery (%) $\boldsymbol{\uparrow}$ | 0.1562 | (-0.0185, 0.3309) | 7.97E-02 |
|  | Perplexity $\boldsymbol{\downarrow}$ | -0.0098 | (-0.0562, 0.0758) | 7.70E-01 |
|  | Loss $\boldsymbol{\downarrow}$ | -0.0295 | (-0.0530, -0.0060) | **1.38E-02** |
| ProtSeqGen vs. GeoSeqBuilder | Recovery (%) $\boldsymbol{\uparrow}$ | 0.6201 | (0.4468, 0.7934) | **6.23E-05** |
|  | Perplexity $\boldsymbol{\downarrow}$ | -0.0878 | (-0.1235, -0.0520) | **9.56E-04** |
|  | Loss $\boldsymbol{\downarrow}$ | -0.0227 | (-0.0383, -0.0071) | **4.40E-03** |

**Supplementary Table 3.** Statistical comparison on TS500 between ProtSeqGen and other methods (mean $\pm$ 95%CI)

| **Comparison** | **Metrics** | **Mean difference** | **95% CI** | **p-value** |
| --- | --- | --- | --- | --- |
| ProtSeqGen vs. ProteinMPNN | Recovery (%) $\boldsymbol{\uparrow}$ | 2.4699 | (2.3726, 2.5672) | **2.68E-12** |
|  | Perplexity $\boldsymbol{\downarrow}$ | -0.1495 | (-0.1715, -0.1275) | **3.15E-07** |
|  | Loss $\boldsymbol{\downarrow}$ | -0.0461 | (-0.0565, -0.0357) | **1.10E-05** |
| ProtSeqGen vs. PiFold | Recovery (%) $\boldsymbol{\uparrow}$ | 0.1510 | (0.0410, 0.2610) | **7.12E-03** |
|  | Perplexity $\boldsymbol{\downarrow}$ | -0.0475 | (-0.0808, -0.0141) | **5.29E-03** |
|  | Loss $\boldsymbol{\downarrow}$ | -0.0043 | (-0.0120, 0.0034) | 2.72E-01 |
| ProtSeqGen vs. GeoSeqBuilder | Recovery (%) $\boldsymbol{\uparrow}$ | 0.0988 | (-0.0483, 0.2460) | 1.88E-01 |
|  | Perplexity $\boldsymbol{\downarrow}$ | -0.0629 | (-0.0933, -0.0324) | **2.91-03** |
|  | Loss $\boldsymbol{\downarrow}$ | -0.0053 | (-0.0121, 0.0015) | 1.28E-01 |

**Supplementary Table 4**. Amino acid composition of native versus ProtSeqGen-designed sequences on TS500

| **Residue type** | **Native** | **Designed** | **Ratio in Native** | **Ratio in Designed** |
| --- | --- | --- | --- | --- |
| **A** | 11624 | 12462 | 0.0882 | 0.0946 |
| **C** | 6561 | 5739 | 0.0498 | 0.0436 |
| **D** | 5676 | 5505 | 0.0431 | 0.0418 |
| **E** | 7949 | 8317 | 0.0603 | 0.0631 |
| **F** | 1598 | 1300 | 0.0121 | 0.0099 |
| **G** | 4812 | 2213 | 0.0365 | 0.0168 |
| **H** | 8831 | 12712 | 0.0670 | 0.0965 |
| **I** | 9764 | 10508 | 0.0741 | 0.0798 |
| **K** | 3031 | 2049 | 0.0230 | 0.0156 |
| **L** | 7601 | 7097 | 0.0577 | 0.0539 |
| **M** | 11990 | 12225 | 0.0910 | 0.0928 |
| **N** | 7315 | 7946 | 0.0555 | 0.0603 |
| **P** | 2860 | 2116 | 0.0217 | 0.0161 |
| **Q** | 5278 | 4565 | 0.0401 | 0.0347 |
| **R** | 6025 | 6265 | 0.0457 | 0.0476 |
| **S** | 7732 | 6829 | 0.0587 | 0.0518 |
| **T** | 7408 | 7327 | 0.0562 | 0.0556 |
| **V** | 1807 | 1352 | 0.0137 | 0.0103 |
| **W** | 4639 | 5147 | 0.0352 | 0.0391 |
| **Y** | 9227 | 10054 | 0.0700 | 0.0763 |

**Supplementary Table 5**. Prediction accuracy for residue types across three secondary structures (H, E, and C) on TS500

| **Residue**  **type** | **Alpha-Helix (H)** | | | **Beta-Sheet (E)** | | | **Coil (C)** | | |
| --- | --- | --- | --- | --- | --- | --- | --- | --- | --- |
|  | Native | Designed | Accuracy | Native | Designed | Accuracy | Native | Designed | Accuracy |
| **A** | 5468 | 4046 | 0.74 | 2054 | 1458 | 0.71 | 3334 | 2434 | 0.73 |
| **C** | 451 | 207 | 0.46 | 554 | 271 | 0.49 | 593 | 279 | 0.47 |
| **D** | 2529 | 1340 | 0.53 | 1129 | 565 | 0.50 | 4291 | 2188 | 0.51 |
| **E** | 4394 | 2636 | 0.60 | 1568 | 988 | 0.63 | 2869 | 1664 | 0.58 |
| **F** | 1795 | 1220 | 0.68 | 1888 | 1247 | 0.66 | 1595 | 1037 | 0.65 |
| **G** | 1678 | 738 | 0.44 | 1577 | 726 | 0.46 | 6509 | 2929 | 0.45 |
| **H** | 948 | 825 | 0.87 | 751 | 593 | 0.79 | 1332 | 1106 | 0.83 |
| **I** | 2696 | 1833 | 0.68 | 3157 | 2021 | 0.64 | 1748 | 1136 | 0.65 |
| **K** | 3026 | 1513 | 0.50 | 1476 | 827 | 0.56 | 2813 | 1463 | 0.52 |
| **L** | 5435 | 3152 | 0.58 | 3387 | 1863 | 0.55 | 3168 | 1774 | 0.56 |
| **M** | 1183 | 745 | 0.63 | 749 | 457 | 0.61 | 928 | 557 | 0.60 |
| **N** | 1544 | 803 | 0.52 | 938 | 469 | 0.50 | 3194 | 1470 | 0.46 |
| **P** | 1159 | 811 | 0.70 | 680 | 442 | 0.65 | 4186 | 2888 | 0.69 |
| **Q** | 2203 | 1212 | 0.55 | 933 | 494 | 0.53 | 1676 | 855 | 0.51 |
| **R** | 2798 | 1483 | 0.53 | 1536 | 768 | 0.50 | 2227 | 1047 | 0.47 |
| **S** | 2315 | 1320 | 0.57 | 1658 | 879 | 0.53 | 3759 | 2067 | 0.55 |
| **T** | 1943 | 836 | 0.43 | 2199 | 990 | 0.45 | 3266 | 1306 | 0.40 |
| **V** | 2752 | 2284 | 0.83 | 4227 | 3339 | 0.79 | 2248 | 1798 | 0.80 |
| **W** | 717 | 466 | 0.65 | 533 | 330 | 0.62 | 557 | 373 | 0.67 |
| **Y** | 1548 | 1084 | 0.70 | 1639 | 1098 | 0.67 | 1452 | 1002 | 0.69 |

**Supplementary Table 6**. Distribution of sample in the IDRome-120 dataset by chain length

| **Length** | **No. of samples** |
| --- | --- |
| 151-200 | 30 |
| 101-150 | 30 |
| 51-100 | 30 |
| < 50 | 30 |

**Supplementary Table 7.** Run time and memory consumption of pretrained models on TS50 and TS500

| **Dataset** | **Method** | **Runtime (s)** | **Memory (GB)** |
| --- | --- | --- | --- |
| TS50 | ProteinMPNN | 1.787 | 1.316 |
|  | PiFold | 1.535 | 1.751 |
|  | GeoSeqBuilder | 2.307 | 1.513 |
|  | **ProtSeqGen** | **1.516** | **1.199** |
| TS500 | ProteinMPNN | 8.711 | 2.755 |
|  | PiFold | 7.603 | 3.463 |
|  | GeoSeqBuilder | 11.138 | 3.285 |
|  | **ProtSeqGen** | **7.421** | **2.410** |

**Supplementary Table 8.** Statistical comparison on IDRome-120 between ProtSeqGen and other methods (mean $\pm$ 95%CI)

| **Comparison** | **Metrics** | **Mean difference** | **95% CI** | **p-value** |
| --- | --- | --- | --- | --- |
| ProtSeqGen vs. ProteinMPNN | Recovery (%) $\boldsymbol{\uparrow}$ | +9.5420 | (9.2913, 9.7927) | **1.95E-14** |
|  | Perplexity $\boldsymbol{\downarrow}$ | -6.0174 | (-6.1857, -5.8491) | **3.42E-14** |
|  | Loss $\boldsymbol{\downarrow}$ | -1.4866 | (-1.5026, -1.4706) | **6.43E-18** |
| ProtSeqGen vs. PiFold | Recovery (%) $\boldsymbol{\uparrow}$ | +6.3046 | (6.1004, 6.5088) | **1.28E-13** |
|  | Perplexity $\boldsymbol{\downarrow}$ | -2.4310 | (-2.4981, -2.3639) | **3.03E-14** |
|  | Loss $\boldsymbol{\downarrow}$ | -0.9828 | (-1.0063, -0.9593) | **8.45E-15** |
| ProtSeqGen vs. GeoSeqBuilder | Recovery (%) $\boldsymbol{\uparrow}$ | +4.8686 | (4.6763, 5.0609) | **7.61E-13** |
|  | Perplexity $\boldsymbol{\downarrow}$ | -2.1135 | (-2.1885, -2.0385) | **2.91E-13** |
|  | Loss $\boldsymbol{\downarrow}$ | -0.8850 | (-0.9007, -0.8693) | **5.68E-16** |

**Supplementary Table 9.** Statistical comparison on TS45 between ProtSeqGen and other methods (mean $\pm$ 95%CI)

| **Comparison** | **Metrics** | **Mean difference** | **95% CI** | **p-value** |
| --- | --- | --- | --- | --- |
| ProtSeqGen vs. ProteinMPNN | Recovery (%) $\boldsymbol{\uparrow}$ | +7.9500 | (7.7713, 8.1286) | **4.78E-15** |
|  | Perplexity $\boldsymbol{\downarrow}$ | -3.3586 | (-3.4053, -3.3120) | **6.30E-17** |
|  | Loss $\boldsymbol{\downarrow}$ | -0.5228 | (-0.5320, -0.5135) | **5.45E-16** |
| ProtSeqGen vs. PiFold | Recovery (%) $\boldsymbol{\uparrow}$ | +1.1980 | (1.0868, 1.3091) | **1.57E-09** |
|  | Perplexity $\boldsymbol{\downarrow}$ | -1.0177 | (-1.0341, -1.0012) | **2.44E-16** |
|  | Loss $\boldsymbol{\downarrow}$ | -0.0402 | (-0.0512, -0.0293) | **1.61E-05** |
| ProtSeqGen vs. GeoSeqBuilder | Recovery (%) $\boldsymbol{\uparrow}$ | +1.3347 | (1.2075, 1.4619) | **1.99E-09** |
|  | Perplexity $\boldsymbol{\downarrow}$ | -0.9717 | (-0.9869, -0.9565) | **1.84E-16** |
|  | Loss $\boldsymbol{\downarrow}$ | -0.0572 | (-0.0680, -0.0464) | **8.05E-07** |

**Supplementary Table 10.** Fine-tuning Performance of ProtSeqGen on IDRome-120 and TS45 (mean $\pm$ SD)

| **Dataset** | **Method** | **Recovery (%)** $\boldsymbol{\uparrow}$ | **Perplexity** $\boldsymbol{\downarrow}$ | **Loss** $\boldsymbol{\downarrow}$ |
| --- | --- | --- | --- | --- |
| IDRome-120 | ProtSeqGen | 41.41 $\pm$ 0.15 | 6.84 $\pm$ 0.03 | 1.92 $\pm$ 0.01 |
|  | **ProtSeqGen-FT-IDR** | **47.89** $\pm$ **0.17** | **6.38** $\pm$ **0.02** | **1.85** $\pm$ **0.01** |
| TS45 | ProtSeqGen | 50.13 $\pm$ 0.08 | 5.09 $\pm$ 0.01 | 1.62 $\pm$ 0.01 |
|  | **ProtSeqGen-FT-FM** | **51.75** $\pm$ **0.07** | **4.93** $\pm$ **0.01** | **1.61** $\pm$ **0.01** |
